# The Ninth International Medical Congress

**Published:** 1887-11

**Authors:** 


### *THE NINTH INTERNATIONAL MEDICAL CONGRESS.*

That the many readers of *The Journal* may know the real character and success of the recent International Medical Congress in Washington, we copy the following leading article from the London *Lancet* of September 24, 1887, pp. 617 and 627. Certainly, no higher or more impartial authority could be invoked. *The Lancet* says:

"The success of the Ninth International Medical Congress is a matter for thankfulness. The interruption of the series of congresses would have been little less than a calamity and a disgrace for the profession in all nations. Any serious imperfection in the meeting, either as respects numbers or the character of the discussions, would have been but little less unfortunate. But the Congress has been held under most honorable auspices; the famous hospitality of the United States has been fully realized; and those who went great distances to attend the Congress have been amply rewarded, and will return to their various countries and duties with higher impressions of their calling and deeper convictions of its progress, both on its scientific and its medical side. We cannot but rejoice that our own country was well represented in many of the Sections; the names of many well-known English physicians and surgeons will have been noticed in the reports which were received by cable from our special correspondents at Washington. We confess that we read the report of the concluding proceedings of the Congress with the most pleasurable emotions, and, not least, the remarks of the English members. A break-down of the Congress in Washington would have been only a less acute pain to us than a break-down in London. And we accept the concluding speeches of our countrymen and of our *confrères* of Berlin and Paris, Dr. Martin and Dr. Landolt, and others, as proof that the Congress has been worthy of its predecessors, that it contained a larger gathering of foreign members than any of them, and that it is calculated to promote the advancement of our art. Those in the United States who have worked to this end, in spite of much discouragement, well deserve the gratitude which was accorded to them by formal resolution. We have purposely abstained, in our allusions to the Congress, from pointedly referring to the domestic differences among our brethren in the States, which threatened to seriously mar the success of the Congress, if not to prevent it altogether. Those who persevered in spite of all opposition, and who have carried through the Congress so successfully, may well be satisfied. They have done a great service to their country and to their profession in all countries. It is not necessary for us to say that they committed no faults and made no mistakes. Such praise is not for mortals in a world so full of 'spilt saltpetre' as ours. But they have carried through the Congress, and we thank them. There is yet one other service they can do: in any official action that now devolves upon them, to strive to obliterate the last relics of discord, and to hand on the light of truth and charity, undimmed and unqualified, to those in Berlin, on whom will now rest the burden of responsibility for the next Congress. They can well afford to be magnanimous, and to help to make the representation of the States at Berlin so complete as to bear no traces of recent divisions.

"The scientific value of the addresses and papers read at the recent Congress cannot be estimated till we have seen them in full. On the whole, judging from abstracts of the papers and discussions, we are inclined to say that they have been practical rather than theoretical, and have had reference to useful rather than transcendental aspects ot medicine. Neither, so far, have we met with much indication of original matter in the papers. But this is no dispraise. It was meet that in the most practical nation in the world papers and discussions should take a practical turn, and deal with questions at their practical point. No branch of medicine can be said to have been neglected, from that which deals with the brain to that which deals with the risks of decayed teeth; including, by the way, several instructive cases of pyæmia. The statistics of vaccination were much advanced in a paper by Dr. Josef Körösi, Director of Communal Statistics of Buda-Pesth, (to which we hope to make more special reference,) and other questions of Public Medicine were made subjects of interesting discussion. Every branch of medicine was well represented—notably the ophthalmic, the gynecological, and the dermatological; so that we venture to forecast that the volumes of the Transaction Reports will have considerable practical importance.

"Notwithstanding that the city of Washington is, like most other cities, liable to suffer an annual desertion by the most influential part of its population during the early autumn, the ninth meeting of the

International Medical Congress has been one of the most successful of its kind. We shall not err in saying that as a social undertaking merely, without regard to its professional aspect, this transatlantic gathering holds a place not second in importance to anything of a similar character that has happened, or is likely soon to happen, on this or that side of the dividing ocean. As an assemblage of medical practitioners and teachers, met together with a common purpose of comparing and correcting the results of observation and invention, of devising new plans for future investigation, and of applying new modes of treatment in the art of healing, its significance is equally remarkable. With the single exception of London, no European capital has attracted so many representatives of medicine and surgery since the international movement first originated. Successful in point of numbers, the conference has not been less successful in possessing in more than one particular a thoroughly representative quality. It has been, in the widest sense, international. Almost every civilized people, besides races which might by courtesy be included in the same category, have contributed towards its membership. The four continents have given of the skill which is available for the treatment of disease in their civil practice, the military and naval services have subscribed each its quota of men and of material for discussion. Again, as regards the subject-matter, it is almost superfluous to point out the illustrative diversities of study by which the sum of medicine can be viewed as through so many windows, and to state that each of these has received its full share of recognition in the working programme of the Congress. Last, but not least, the whole business and pleasure of this meeting have been administered with a cordial good fellowship truly representative of that spirit of hospitality which happily characterizes the American people, and annually admits to the privileges of free citizenship strangers out of every country without distinction. President Cleveland and his assessors in the work of government have been foremost in showing attention to the American practitioners and their guests. Not only have they taken an active part in the extra professional work of the Congress, but it is through their influence that government buildings have been on several occasions available for social gatherings of the members. We are happy to recall the fact that in so doing they have but revived the precedents afforded by the conduct of other States, but we appreciate no less on that account the warmth of their generous consideration. Nor must we forget to offer an equal tribute of esteem to the American medical press, and to those citizens of Washington who distinguished themselves by affording a similarly handsome hospitality."

Inasmuch as the International Medical Congress of Washington has passed and been freely awarded a high and honorable position with its predecessors by the medical press of other countries, we desire to add only an earnest hope that the medical press of this country will accept the gratifying result, and instead of nursing old prejudices or seeking for imperfections and errors, to which all human beings are liable, will henceforth labor for the unity and advancement of the profession as a whole, and to secure an American representation in the International Medical Congress of 1890, at Berlin, as intelligent and honorable as came from the other side of the Atlantic to Washington in the Congress of 1887.—*American Medical Journal.*

